# Prevalence of *Plasmodium ovale* and *Plasmodium malariae* Mixed Infections and Associated Mortality in Children with Severe *Falciparum* Malaria

**DOI:** 10.4269/ajtmh.25-0514

**Published:** 2026-04-16

**Authors:** Giselle Lima-Cooper, Dibyadyuti Datta, Caitlin Bond, Kagan A. Mellencamp, Ruth Namazzi, Mahesh Gupta, Andrea L. Conroy, Robert O. Opoka, Chandy C. John

**Affiliations:** ^1^ Ryan White Center for Pediatric Infectious Diseases and Global Health, Department of Pediatrics, Indiana University School of Medicine, Indianapolis, Indiana, USA;; ^2^ Department of Paediatrics and Child Health, Makerere University, Kampala, Uganda;; ^3^ University of Michigan Medical School, Ann Arbor, Michigan, USA;; ^4^ Aga Khan University, Nairobi, Kenya

## Abstract

The prevalence and clinical significance of mixed *Plasmodium* infections in children with severe *Plasmodium falciparum* (*P. falciparum*) malaria are not well characterized. In a cohort of 440 Ugandan children hospitalized with severe malaria who were diagnosed with *Plasmodium* species via microscopy, nested polymerase chain reaction (nPCR) testing was used to detect *P. falciparum*, *Plasmodium ovale* (*P. ovale*), *Plasmodium malariae* (*P. malariae*), and *Plasmodium vivax* (*P. vivax*) infections and assess their association with adverse clinical outcomes during hospitalization. Using nPCR testing, the *Plasmodium* *18S* small-subunit ribosomal RNA gene was detected in 440 children. *Plasmodium falciparum* mono-infection was identified in 329 (74.8%) children, *P. malariae* mono-infection was detected in one (0.2%), *P. falciparum* and *P. ovale* mixed infections were detected in 100 (22.8%), *P. falciparum* and *P. malariae* mixed infection was detected in one (0.2%), and *P. falciparum*, *P. ovale*, and *P. malariae* triple infections were detected in nine (2.1%). Children with triple infections of *P. falciparum*, *P. ovale*, and *P. malariae* exhibited higher mortality rates than those with *P. falciparum* mono-infection (3/9 [33%] versus 23/329 [7%]; odds ratio = 7.0; 95% CI = 1.8–28; *P* = 0.005). They were also more likely to experience respiratory distress (6/9 [67%] versus 115/329 [35%]; *P* = 0.08). In the present study, a high proportion of children with severe *falciparum* malaria had mixed *P. ovale* infections, and *P. falciparum*, *P. ovale*, and *P. malariae* triple infections were associated with increased mortality. Improved detection and broader surveillance of non-*falciparum* malaria may help identify epidemiologic patterns associated with adverse outcomes in severe malaria.

## INTRODUCTION

*Plasmodium falciparum* (*P. falciparum*) is the predominant species responsible for malaria-related morbidity and mortality in children, accounting for more than 99% of human malaria infections.^1^^,^^2^ However, non-*falciparum Plasmodium* species, including *Plasmodium malariae* (*P. malariae*), *Plasmodium ovale* (*P. ovale*), and *Plasmodium vivax* (*P. vivax*), are also widely present in malaria-endemic areas.^3^^–^^6^ Severe malaria cases caused by *P. malariae* or *P. ovale* mono-infections have been reported, particularly in immunocompromised individuals, blood transfusion recipients,^7^^–^^12^ travelers returning from endemic areas,^13^ and children with *P. malariae*-associated nephrotic syndrome.^14^^,^^15^ In Uganda, non-*falciparum* species have been detected in children with uncomplicated malaria^3^ and blood donors^16^ using sensitive detection methods such as nested polymerase chain reaction (nPCR) testing. However, the impact and burden of mixed *Plasmodium* infections in African children with severe malaria are poorly understood. In the field, multispecies infections in children with severe malaria can be difficult to detect because most cases are diagnosed using *P. falciparum-*specific single-antigen rapid diagnostic tests (RDTs) or microscopy, which may miss other species because of the higher peripheral parasite densities in *P. falciparum* compared with non-*falciparum* species.^17^

Understanding the prevalence and impact of *Plasmodium* mixed infections in children with severe *falciparum* malaria is crucial because mixed infections may exacerbate disease severity and increase the risk of adverse outcomes in an already high-risk population. Although mixed infections have been studied in the context of asymptomatic or uncomplicated malaria,^3^^,^^5^^,^^17^^–^^19^ their role in severe malaria in children is unclear. To address this gap, samples from children with severe *falciparum* malaria enrolled in a prospective cohort study at two geographically distinct sites in Uganda were analyzed. Using nPCR testing, the prevalence of *P. falciparum*, *P. ovale*, *P. malariae*, and *P. vivax* infections and their association with adverse outcomes during hospitalization were assessed.

## MATERIALS AND METHODS

### Study population.

A prospective study was conducted between 2014 and 2017 to assess cognition 12 months after hospital discharge in 600 children aged 6 months to 4 years diagnosed with severe *P. falciparum* malaria via RDT or microscopy.^20^ Children diagnosed with severe malaria via microscopy (*N* = 440) are included in the present study to enable a direct comparison of the detection of multiple *Plasmodium* species infection with nPCR testing. The present study was performed in Kampala, an urban city with low malaria transmission, and Jinja, a peri-urban region with moderate malaria transmission.^20^^,^^21^ Severe malaria was defined on the basis of diagnostic evidence through direct visualization of malaria parasites via microscopy in children hospitalized with one or more of the five most common WHO criteria for severe malaria: coma (Blantyre Coma Score <3), respiratory distress (deep acidotic breathing or lower chest wall retractions), seizures (two or more in 24 hours), severe anemia (hemoglobin ≤5 g/dL), or prostration (inability to sit unsupported or stand in children ≥1 year old or unable to breastfeed in children <1 year old).^22^ Bacteremia was assessed via blood culture in all children with sufficient whole blood, as described previously.^21^ Blackwater fever was identified on the basis of parental report of tea- or “Coca-Cola”-colored urine, which has been shown to correlate with 80% of urine dipstick results for hemoglobin.^21^^,^^23^ Malaria parasite biomass was assessed via ELISA to quantify plasma *Plasmodium falciparum* histidine-rich protein 2 (*Pf*HRP2) levels using Malaria Ag CELISA (Cellabs, Brookvale, Australia).^21^ The children were treated according to the Ugandan National Treatment guidelines, which include intravenous artesunate, followed by oral artemether–lumefantrine. Detailed inclusion and exclusion criteria, as well as the clinical care of the study participants, have been described previously.^20^

### Study procedures and clinical laboratory testing.

After enrollment, children underwent a comprehensive history interview and physical examination, with clinical complications assessed in the following organ systems: cerebral (coma, multiple seizures), respiratory (respiratory distress, deep breathing, hypoxia), hematological (severe anemia [hemoglobin ≤5 g/dL], thrombocytopenia [platelet count <150 × 10^3^/*µ*L]), kidney (acute kidney injury, defined as a 1.5-fold increase in serum creatinine from estimated baseline or 0.3 mg/dL increase in creatinine within 24 hours of admission), metabolic (acidosis [base excess ≤8 mmol/L, or bicarbonate <15 mmol/L if base excess was missing, or lactate >5 mmol/L if base excess and bicarbonate were missing], hypoglycemia [glucose <2.2 mmol/L on admission], hyperkalemia [potassium ≥6.0 mmol/L]), and hepatic (jaundice, hypoalbuminemia [albumin <3.5 mg/dL]).^20^ Biomarkers of intestinal injury were analyzed in serum or plasma from all cryopreserved samples by measuring trefoil factor 3 (TFF3) and intestinal fatty acid binding protein (I-FABP) using a custom Luminex MagPix (Luminex Corporation, Austin, TX) panel, as described previously.^24^ Children were considered to have intestinal injury if either their TFF3 or I-FABP levels were elevated relative to population reference levels (with >99th percentile of the population used as the upper limit of normal).^24^

Giemsa-stained peripheral blood smears were examined via light microscopy to detect *Plasmodium* infection, with independent readings performed by two microscopists. A smear result was considered positive if both the initial microscopists reported a positive reading. Parasite density was calculated as the mean value of two readings unless there was a density discrepancy of >20%; in this case, a third reading was obtained, and the median of the three values was used. Parasites were identified on the basis of 200–500 white blood cells (WBCs) on thick smears and species on thin smears. The patient’s WBC count from a complete blood count was used to calculate parasite density per microliter.

### *Plasmodium* species detected via nPCR testing.

Whole blood samples were collected in ethylenediaminetetraacetic acid, sodium citrate, or lithium heparin tubes, stored at −20°C, and transported to the United States on dry ice. Upon arrival, the samples were stored at −80°C until processing. Genomic DNA was isolated using the QIAamp DNA Blood Mini Kit (Qiagen, Hilden, Germany), according to the manufacturer’s instructions. Nested polymerase chain reaction targeting the *18S* small subunit (SSU) ribosomal RNA (rRNA) gene was used for *Plasmodium* spp. amplification.^25^^–^^27^ Briefly, in nPCR-1, genus-specific primers rPLU1 and rPLU5^25^^,^^26^ were used for *Plasmodium* spp. amplification, followed by nPCR-2, which was conducted using species-specific primers for rFAL1 and rFAL2, rMAL1 and rMAL2, rVIV1 and rVIV2, rOva1 and rOVA2,^26^ and rOVA1 and rPLU2.^25^ Nested polymerase chain reaction-1 was performed in a 20 *µ*L reaction volume containing HotStar Taq Master Mix (Qiagen), 0.2 *µ*M of forward and reverse primers, and 4 *µ*L of DNA. For nPCR-2, 1 *µ*L of the nPCR-1 product was added to 20 *µ*L of the reaction mix. Both polymerase chain reaction (PCR) steps were performed with the same cycling parameters: incubation at 95°C for 5 minutes, followed by 35 cycles at 95°C for 30 seconds, incubation at 58°C for 1 minute, and a final incubation at 72°C for 1 minute.^27^ Positive controls included genomic DNA from *P. falciparum* strain 3D7 (MRA-102), *P. vivax* strain Chesson (MRA-383), a *P. ovale* strain (MRA-180), and a diagnostic plasmid containing the SSU rRNA gene for a *P. malariae* strain (MRA-179) and *P. vivax* (MRA-178), obtained from the European Malaria Reagent Repository and BEI Resources, National Institute of Allergy and Infectious Diseases, NIH. Negative controls consisted of nuclease-free water and DNA from donors who had not been exposed to malaria.

### *Plasmodium* species confirmation using Sanger sequencing.

*Plasmodium* spp. amplicons from purified nPCR were further confirmed using Sanger sequencing (Quintara Bioscience, Boston, MA) and queried against the BLAST database (National Center for Biotechnology Information, Bethesda, MD).

## STATISTICAL ANALYSES

The characteristics of the study participants were compared using the Wilcoxon rank-sum test for continuous measures and Pearson’s χ^2^ test or Fisher’s exact test for categorical measures. Logistic regression with Firth’s penalized likelihood method was used to evaluate the association between infection groups of *Plasmodium* species and in-hospital mortality, given the small number of deaths and the potential for separation. Pairwise comparisons of mortality across the three infection groups yielded three tests. *P*-values for these comparisons were adjusted using the Bonferroni correction (α = 0.05/3 = 0.017); statistical significance was defined as *P* <0.017. All other analyses were exploratory or secondary and were not adjusted for multiple tests. All statistical analyses were performed using Stata version 18.0SE (StataCorp LLC, College Station, TX).

## RESULTS

### Detection of *P. falciparum* parasitemia via microscopy and nPCR.

**Figure 1.**
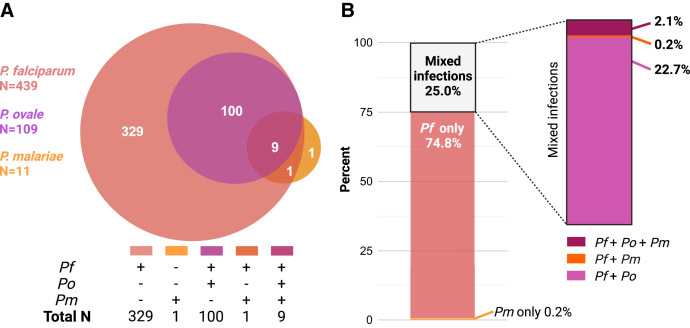
Prevalence of *Plasmodium falciparum*, *Plasmodium ovale*, and *Plasmodium malariae* according to nested polymerase chain reaction (nPCR) testing in children with severe malaria who tested positive for *Plasmodium* species by microscopy (*N* = 440). (**A**) Venn diagram illustrating the number of children with severe malaria in whom *Plasmodium* species were identified via nPCR, including mono- and mixed-species infections (*N* = 440). *Plasmodium* species in mono-infections and mixed infections identified by color. (**B**) Proportion of *Plasmodium* infections detected via nPCR in children with severe malaria. The vertical axis represents the proportions of mono-infections and mixed infections. Colored bars represent species.

In the present study, 440 children with severe malaria tested positive for *Plasmodium* parasites by microscopy (*P. falciparum* [*n* = 437], *P. ovale* [*n* = 1], and *P. malariae* [*n* = 2] mono-infections; no mixed infections were detected) and were included in the present analysis. Among the 440 children, 439 (99.8%) tested positive for *P. falciparum* by nPCR testing, and one child (0.2%) tested positive for *P. malariae* mono-infection by nPCR (Figure 1A).

### Mixed *Plasmodium* spp. infections are common in children with severe *P. falciparum* malaria.

Of the 439 children with severe malaria who tested positive for *P. falciparum* by nPCR, 74.8% (*n* = 329) had *P. falciparum* mono-infection, 0.2% (*n* = 1) had *P. malariae* mono-infection, and 25.0% (*n* = 110) had mixed *P. falciparum* infections, with 22.7% (*n* = 100) testing positive for *P. falciparum* and *P. ovale*, 0.2% (*n* = 1) testing positive for *P. falciparum* and *P. malariae*, and 2.1% (*n* = 9) testing positive for triple infections with *P. falciparum*, *P. ovale*, and *P. malariae* (Figure 1A and B). No *P. vivax* infection was detected. Among the samples that tested positive for *P. ovale* or *P. malariae*, Sanger sequencing was performed bidirectionally on 37% (41/110) of the mixed infection samples, yielding the following results: *P. falciparum* and *P. ovale* (*n* = 31); *P. falciparum*, *P. ovale*, and *P. malariae* (*n* = 9); and *P. malariae* (*n* = 1). BLAST analysis confirmed species identification in all samples, with matching criteria of E-value ≤1e-5, query cover >70%, and identity match >70%.

### Demographic and clinical characteristics of children with mixed-species malaria diagnosed via nPCR.

The demographic and clinical characteristics of children with *P. falciparum* mono-infections and mixed *P. falciparum* infections are described in Table 1. Among children with severe malaria, peripheral parasite density was significantly higher in those with *P. falciparum* mono-infection than in those with mixed *P. falciparum* infections (*P* <0.01). However, no difference in *Pf*HRP2 levels was observed between the two groups (*P* = 0.23). Platelet counts were lower in children with *P. falciparum* mono-infection than in those with mixed *P. falciparum* infections (*P* = 0.01). Similar demographic and clinical characteristics were observed in both groups, including age, sex, study site, hemoglobin levels, and WBC counts. Notably, one child diagnosed with *P. falciparum* infection via microscopy, with a parasite density of 11,393 parasites/*µ*L (Table 1), was found to have *P. malariae* mono-infection by nPCR.

**Table 1:** Enrollment characteristics by the presence of *Plasmodium falciparum* mono-infection or mixed infection detected via nested polymerase reaction testing in children with severe malaria diagnosed by microscopy, *N* = 440

	*P. falciparum* Only by nPCR + (*n* = 329)	Mixed *P. falciparum* or non-*P. falciparum* by nPCR (*n* = 111)*	*P*-Value^†^
Age, years	2.0 (1.3–2.8)	2.0 (1.5–2.9)	0.67
Sex, female, *n* (%)	145 (44.1)	49 (44.1)	0.99
Site			
Kampala, *n* (%)	183 (55.6)	59 (53.1)	0.69
Jinja, *n* (%)	146 (44.4)	52 (46.9)	–
Peripheral parasite density, parasites/*µ*L	64,319 (8,726–249,278)	23,799 (2,474–143,220)	**<0.01**
Plasma *Pf*HRP-2 levels, ng/mL	3,528 (1,364–7,365) (326)	2,883 (1,153–5,828) (110)	0.23
Hemoglobin, g/dL	6.5 (4.1–8.9) (328)	6.1 (3.8–8.7) (110)	0.81
Platelet count (×10^3^/*µ*L)	89.6 (50–158) (328)	113.5 (62–209) (110)	**0.02**
WBC count (×10^3^/*µ*L)	11.1 (8.3–17.4) (328)	12.1 (9.0–18.0) (110)	0.30
Glucose, mmol/L	5.8 (4.6–7.2) (302)	5.7 (3.8–7.1) (102)	0.42
Lactate, mmol/L	4.3 (2.5–8.1) (312)	4.5 (3.0–7.0) (109)	0.45
Serum creatinine, mg/dL	0.4 (0.3–0.5) (329)	0.4 (0.3–0.5) (110)	0.83
Potassium, mmol/L	4.1 (3.7–4.5) (307)	4.2 (3.8–4.5) (103)	0.30

HRP-2 = *Plasmodium falciparum* histidine-rich protein; nPCR = nested polymerase chain reaction; *P. falciparum* = *Plasmodium falciparum*; WBC = white blood cell. All values are reported as median (interquartile range), except where indicated. For variables for which *n* is less than the total *N* listed for the group, the numbers for that variable and group are noted in the table. For these secondary analyses, *P*-values were not adjusted for multiple comparisons. *P*-values <0.05 are highlighted in bold.

* One child identified as having a *P. falciparum* infection by microscopy was positive for *Plasmodium malariae* only on nPCR.

^†^ The Wilcoxon rank-sum test was used to compare continuous measures on the basis of *P. falciparum* nPCR results. The χ^2^ test or Fisher’s exact test was used to compare categorical outcomes on the basis of nPCR results, as appropriate.

### Distribution of mixed infections during the study period (2014–2017).

**Figure 2.**
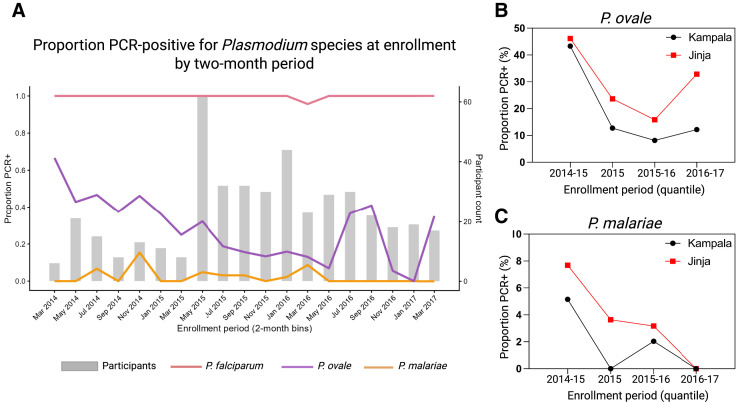
Proportion of *Plasmodium* species detected via nested polymerase chain reaction (PCR) testing among severe malaria cases during the study period (2014–2017). (**A**) Proportion of enrollment samples positive for *Plasmodium falciparum*, *Plasmodium ovale* (*P. ovale*), and *Plasmodium malariae* (*P. malariae*) by nested PCR, aggregated into 2-month enrollment intervals. Bars indicate the number of participants enrolled per interval (right *y* axis). Proportion of (**B**) *P. ovale* and (**C**) *P. malariae* PCR-positive cases by enrollment period (quantiles), stratified by study site (Kampala and Jinja).

The proportion of children with mixed *P. falciparum* and *P. ovale* infections detected via nPCR was similar at both sites (Kampala: 22% [53/242] and Jinja: 24% [47/198]), as was the proportion of mixed infections with *P. falciparum*, *P. ovale*, and *P. malariae* (Kampala: 2% [5/242], Jinja: 2% [4/198]; *P* = 0.65 and *P* = 0.62, respectively; Supplemental Table 1). However, there was a notable fluctuation in the proportion of *P. ovale* infections, with a decrease in 2015, followed by an increase between May and September 2016, and a second increase in March 2017 (Figure 2A). In Kampala, the proportion of *P. ovale* infections decreased over time (Figure 2B). For *P. malariae*, the proportion of infections decreased over time across both sites (Figure 2C).

### Triple infections with *P. falciparum*, *P. malariae*, and *P. ovale* are associated with increased mortality in severe malaria.

**Figure 3.**
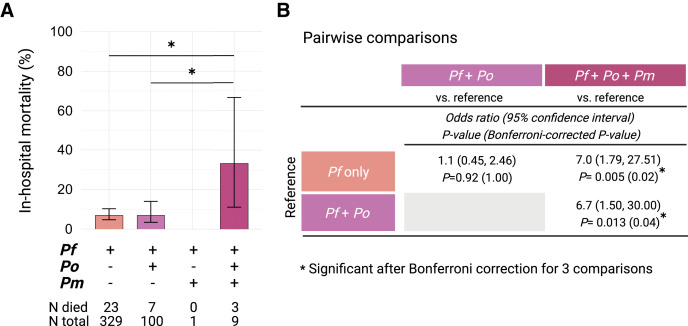
In-hospital mortality rates of children with severe malaria who were positive for *P. falciparum* by microscopy, according to *Plasmodium* species defined by nested polymerase chain reaction (nPCR) testing, including mono-infections and mixed infections with *Plasmodium falciparum* (*P. falciparum*), *Plasmodium ovale*, and *Plasmodium malariae* (*P. malariae*). (**A**) Colored bars and error bars represent the proportion of children who died in the hospital and the 95% CIs for each nPCR group. The number who died and the total number in each group are displayed under the graph. (**B**) Pairwise comparisons from binary logistic regression with penalized maximum likelihood. *P*-values from pairwise comparisons are shown before and after Bonferroni correction for three comparisons. Children with a *P. malariae* mono-infection (*n* = 1) or mixed *P. falciparum* and *P. malariae* infections (*n* = 1), both of whom survived, were excluded from the analysis.

Among the nine children with severe malaria who had mixed *P. falciparum, P. ovale*, and *P. malariae* infections, three (33%) died (Figure 3A). One death occurred in Kampala, and two occurred in Jinja. Among children with *P. falciparum* mono-infection, 7% (23/329) died (Figure 3A). Compared with children with *P. falciparum* mono-infections, children with *P. falciparum, P. ovale*, and *P. malariae* triple infections had significantly higher odds of death (odds ratio [OR] = 7.0; 95% CI = 1.79–27.51; *P* = 0.005; Bonferroni-adjusted *P* = 0.02). Compared with children with mixed *P. falciparum* and *P. ovale* infections, children with *P. falciparum, P. ovale*, and *P. malariae* triple infections had 6.7-fold increased odds of death (95% CI = 1.50–30.00; *P* = 0.013; Figure 3B). Mortality in children with mixed *P. falciparum* and *P. ovale* infections (7.0%) did not differ from *P. falciparum* mono-infection (OR = 1.1; 95% CI = 0.45–2.46; *P* = 0.92; Figure 3B).

### Clinical complications in children with severe malaria and *Plasmodium* triple infections compared with *P. falciparum* mono-infections and mixed *P. falciparum* and *P. ovale* infections.

Descriptive comparisons of the sociodemographic factors, nutritional factors, fever duration, and clinical factors among children with severe malaria and triple infections (*P. falciparum, P. ovale*, and *P. malariae*) versus children with *P. falciparum* mono-infection or mixed *P. falciparum* and *P. ovale* infection are detailed in Supplemental Table 2. Sociodemographic factors, nutritional factors, and fever duration did not differ between the groups. Clinical and laboratory indicators of disease severity were similar between children with *P. falciparum* mono-infections and those with *P. falciparum* mixed infections (Supplemental Table 2). Respiratory complications were observed in 6/9 (67%) children with *P. falciparum*, *P. malariae*, and *P. ovale* triple infections compared with 115/329 with *P. falciparum* mono-infections (35%) and 29/100 with mixed *P. falciparum* and *P. ovale* infections (29/100 [29%]; *P* = 0.08 and *P* = 0.03, respectively, not adjusted for multiple comparisons; Supplemental Table 2). Bacteremia was rare in children with severe malaria (3%), and none of the eight children with triple infections (*P. falciparum*, *P. ovale*, and *P. malariae*) had bacteremia (Supplemental Table 3).

## DISCUSSION

In Uganda, where *P. falciparum* infections account for the majority of severe malaria cases, the present study reveals a high prevalence of mixed *P. ovale* infections in children with severe *falciparum* malaria and a substantial burden of mixed-species infections in hospitalized children with severe *falciparum* malaria. Nearly 25% of children had mixed infections with *P. ovale*, or with both *P. ovale* and *P. malariae* detectable only by nPCR, emphasizing the limitations of microscopy in detecting non-*falciparum* species, particularly when *P. falciparum* is present. A higher risk of in-hospital mortality was observed in children with triple-species infections (*P. falciparum, P. ovale*, and *P. malariae*) than in children with *P. falciparum* mono-infection or mixed *P. falciparum* and *P. ovale* infections. These findings raise questions regarding the clinical relevance of non-*falciparum* species in severe malaria and highlight the need for improved diagnostic strategies beyond *P. falciparum* detection to inform treatment and prognosis in malaria-endemic settings.

According to nPCR testing, 75% of severe malaria cases were caused by *P. falciparum* mono-infection, whereas 25% were caused by *P. falciparum* infections mixed with *P. ovale* or *P. malariae*. These findings are consistent with existing evidence that *P. falciparum* is the leading driver of severe malaria in African children. However, the finding that 25% of children with severe *P. falciparum* malaria had mixed infections, most commonly with *P. ovale*, highlights a significant burden of mixed-species infections in African children. The data are consistent with reports from travelers and migrants from Africa, where severe malaria was observed in 21% (8/38) of mixed *P. falciparum* infections, compared with 10% of *P. falciparum* mono-infection (146/1,548), 5% of *P. ovale* mono-infection (10/188), and 3% of *P. malariae* mono-infection (2/61).^13^ The low prevalence of *P. ovale* or *P. malariae* mono-infections in children with severe malaria is consistent with studies from Uganda^3^ and Namibia,^28^ in which 90–100% of symptomatic or asymptomatic *P. ovale* infections in children, detected via nPCR, were mixed infections with *P. falciparum*. In contrast, a recent study in Tanzania revealed a high prevalence of *P. ovale* mono-infection (15%), detected via PCR, in asymptomatic schoolchildren.^29^

Mortality was substantially higher among children with triple infections compared with those with *P. falciparum* mono-infection and mixed *P. falciparum* and *P. ovale* infections, with a sevenfold increase in the odds of death for patients with triple infections compared with those with *P. falciparum* mono-infection. Because no mixed-species infections were evident by microscopy, these infections would have gone undetected without molecular diagnosis. Overall, these findings are consistent with *P. falciparum* being identified as the main cause of global malaria mortality,^30^ as well as low mortality rates for *P. ovale* or *P. malariae* mono-infections.^7^^,^^31^^–^^33^ The lack of data on the mortality risk associated with mixed *Plasmodium* infections in Africa is likely influenced by the predominance of microscopy- and RDT-based *P. falciparum* detection. Furthermore, standard care protocols rely on prompt treatment once *P. falciparum* infection is detected, and additional species detection is not often performed. The progression of an infection may be affected by the simultaneous presence of multiple *Plasmodium* species in a mixed infection^34^^,^^35^ and may reflect competition among parasites or more frequent complications, including severe anemia, renal impairment, and pulmonary complications, which may impact outcomes. Consistent with this finding, the present study revealed a trend toward increased respiratory distress among children with triple infections. *Plasmodium malariae* and *P. ovale* may increase the risk of respiratory distress via anemia, inflammatory cytokines, and pulmonary edema.^9^^,^^12^^,^^36^^,^^37^ Therefore, increased respiratory complications may explain the increased mortality in children with severe malaria and multiple *P. falciparum, P. malariae*, and *P. ovale* species infections. This finding is consistent with reports of an increased proportion of multiple-organ failure associated with *P. falciparum* mixed infections compared with *P. falciparum* mono-infection; common complications included severe anemia, pulmonary failure, and renal impairment.^35^ However, other confounding factors that increase mortality may also be associated with triple infections with *P. falciparum*, *P. malariae*, and *P. ovale*. Although no differences in socioeconomic status or nutritional parameters were found between children with triple infections and those with *P. falciparum* mono-infection or mixed *P. falciparum* and *P. ovale* infections, the association between triple infection and increased mortality could be due to other unmeasured confounding factors. Interestingly, platelet count was increased in children with multiple infections or non-*P. falciparum* infection, >95% of whom had mixed *P. falciparum* and *P. ovale* infections or mixed *P. falciparum*, *P. ovale*, and *P. malariae* infections. A low platelet count is strongly associated with higher mortality in severe *P. falciparum* malaria;^38^ thus, the finding of an increased platelet count in those with multiple *Plasmodium* species infections was unexpected. In Southeast Asia, coinfection with *P. vivax* attenuates disease severity in individuals with *P. falciparum* infection.^39^ These contrasting findings could indicate that the infection response is more closely related to the host than the parasite, and this area requires further study.

Given the limited sample size of *Plasmodium* triple infections, the present study was underpowered to identify more subtle differences in complications or pathways associated with severe malaria pathogenesis. Addressing these questions would require longitudinal analysis and a larger sample to support causal inference. Although meta-analyses have been conducted to examine mixed-species infections and mortality in adults, no eligible studies from Africa were found,^35^ and the reported mixed infections were most often *P. vivax* infections. Thus, the present study offers unique insights into severe malaria outcomes among African children with mixed-species infections. The study findings are consistent with those of studies in adults and experimental models of mixed *Plasmodium* infections. In murine models, mixed-species infections are more virulent, with 100% mortality reported in mixed infections compared with 40% in mono-infections.^40^

The present study (2014–2017) revealed a high proportion (23%) of mixed *P. falciparum* and *P. ovale* infections in children with severe malaria, compared with 3% reported in a 2016 surveillance study of mixed malaria infections across 10 sites in children with symptomatic malaria.^3^ This suggests that mixed infections may be more common in severe malaria than uncomplicated malaria; however, these findings must be confirmed in larger cohorts. Although no significant differences in the proportions of non-*falciparum* species were observed between sites in the present study, reports have revealed regional differences in non-*falciparum* prevalence across Uganda, where indoor residual spraying has been implemented to interrupt *P. falciparum* transmission.^3^ In the present study, no *P. vivax* cases were identified, which is consistent with previous reports revealing that *P. vivax* is rare in Uganda.^3^^,^^41^^,^^42^ *Plasmodium vivax* cases are particularly uncommon among individuals with a Duffy-negative phenotype in East Africa,^43^ although infections can still occur in these individuals.^44^ However, *P. vivax* infections are more prevalent in neighboring Ethiopia, highlighting the need for technicians to be trained to recognize *P. vivax* cases in epidemiological investigations. The study findings align with recent reports from Central and Eastern Africa, where the prevalence of non-*falciparum* infections varies. Higher rates of *P. malariae* have been observed in Central and Western Africa (8–15%).^45^^–^^49^ In comparison, *P. ovale* is more common in Eastern and Southern Africa (12–24%),^29^^,^^50^^,^^51^ with non-*falciparum* infections often occurring as mono-infections or mixed infections with *P. falciparum*. Data on mixed infections^18^^,^^19^ suggest that mixed *P. falciparum* and *P. ovale* infections are common. The proportion of *P. ovale* infections can fluctuate, which is consistent with the variable *P. ovale* infection rates observed in the present study. For example, a study in Kenya revealed an increase in the prevalence of *P. ovale* among symptomatic malaria cases between 2008 and 2016.^50^ Together, these findings suggest a much higher burden of non-*falciparum* infections than is currently reflected in WHO malaria reports, with results consistent across geographical regions and populations. This emphasizes the importance of using accurate diagnostic tools to detect non-*falciparum* species in Africa and highlights the need for enhanced surveillance to monitor regional heterogeneity in prevalence and temporal trends, particularly when malaria prevention programs are implemented. In the present study, the risk of malaria recurrence with concurrent *P. ovale* infection in children with severe *P. falciparum* malaria was not evaluated; however, this is an aim for future studies. Diagnosis of *P. ovale* malaria would require treatment with primaquine, in addition to standard treatment of severe *P. falciparum* malaria, to eradicate *P. ovale* hypnozoites that can lead to recurrent infection.

The key strengths of the present study include the enrollment of participants from two geographically distinct sites with varying malaria transmission rates, as well as a thorough and rigorous characterization of severe malaria. Differences in the proportion of *P. ovale* across sites, over time, and by season further support the need for wider surveillance. Although the study cohort of 440 children with severe malaria was relatively large, the parent study was not designed to detect species-level differences in mortality. Notwithstanding, differences in mortality were found between *P. falciparum* mono-infections and triple infections (*P. falciparum, P. malariae*, and *P. ovale*) using a conservative statistical approach that accounted for multiple comparisons. These findings highlight an overlooked area of surveillance that warrants further validation across additional sites and larger populations. Although differences in mortality were observed between children with triple infections and those with *P. falciparum* mono-infections, the small number of events and the cross-sectional study design limited the authors’ ability to draw definitive conclusions regarding the clinical impact of mixed infections on mortality. Longitudinal studies in multiple malaria-endemic areas are needed to confirm whether the presence of multispecies infections is common in children with severe malaria throughout African malaria-endemic regions, as well as whether triple infections, if present in these areas, are associated with increased mortality.

## CONCLUSION

The present study reveals that non-*falciparum* species are commonly found in Ugandan children with severe *P. falciparum* malaria. Additionally, mixed infections with *P. falciparum*, *P. ovale*, and *P. malariae* in children with severe malaria were associated with increased odds of mortality compared with *P. falciparum* mono-infection in this cohort. These findings emphasize the need for additional testing across different African regions to better understand the prevalence of mixed infections in severe malaria and their potential impact on disease severity and mortality. Finally, the present study highlights an urgent need for more sensitive and cost-effective diagnostic tools for detecting non-*falciparum Plasmodium* species, which are often missed by standard microscopy and RDT methods.

## Supplemental Materials

10.4269/ajtmh.25-0514 Supplemental Materials
