# Supplementary material for: Prevalence of Plasmodium ovale and Plasmodium malariae Mixed Infections and Associated Mortality in Children with Severe Falciparum Malaria
Source: Am J Trop Med Hyg. 2026 Apr 16;114(6):1120–7. doi: 10.4269/ajtmh.25-0514 (PMC13235610; doi:10.4269/ajtmh.25-0514)
Supplement: Supplemental Materials [file tpmd250514.SD1.pdf]

Table S1. Prevalence of *P. falciparum*, *P. malariae, and P. ovale* by nPCR in children with severe malaria, according to study site

			Severe malaria	
Pf	***Po***	***Pm***	Kampala (N=242)	Jinja (N=198)
**+**	**-**	**-**	183 (76)	146 (74)
**-**	**+**	**-**	0	0
**-**	**-**	**+**	1 (0.4)	0
**+**	**+**	**-**	53 (22)	47 (24)
**+**	**-**	**+**	0	1 (0.5)
**+**	**+**	**+**	5 (2)	4 (2)

Values are presented as n (%). Abbreviations: nPCR, nested polymerase chain reaction*; Pf, Plasmodium falciparum*; *Pm*, *P. malariae; Po, P. ovale.*

Table S2. Demographic, clinical and laboratory markers in children with severe malaria and triple-infection with *Plasmodium falciparum (Pf)*, *P. ovale (Po)*, and *P. malariae (Pm)* compared to *Pf* mono-infection or *Pf* and *Po* mixed infection

	***Pf* + *Po* + *Pm* (n=9)**	**vs. *Pf* only (n=329)**	***P*-value**	**vs. *Pf* + *Po* (n=100)**	***P*-value**
**Sociodemographic factors**					
Socioeconomic status, median (IQR)	8 (7, 12) (*n=6*)	11 (8, 14) (*n=272*)	0.15	10 (8,14) (*n=86*)	0.09
Fever duration, days, median (IQR)	3 (2, 6) (*n=7*)	3 (2, 4) (*n=327*)	0.56	3 (2, 5)	0.69
**Nutritional factors**					
Weight-for-age z-score, median (IQR)	-1.1 (-1.9, -0.7)	-0.9 (-1.6, -0.3)	0.61	-1.1 (-1.9, -0.2)	0.78
Height-for-age z-score, median (IQR)	-1.8 (-2.2, -0.3)	-1.0 (-1.8, -0.2)	0.34	-1.2 (-1.9, -0.5)	0.62
Weight-for-height z-score, median (IQR)	-0.4 (-1.1, 0.6)	-0.6 (-1.4, 0.1)	0.48	-0.8 (-1.3, 0.1)	0.48
**Cerebral complications**	3 (33.3)	159 (48.3)	0.51	53 (53.0)	0.31
Multiple seizures	2 (22.2)	150 (45.6)	0.19	48 (48.0)	0.18
Coma	3 (33.3)	43 (13.1)	0.11	16 (16.0)	0.19
**Respiratory complications**	6 (66.7)	115 (35.0)	0.08	29 (29.0)	**0.03**
Respiratory distress	5 (55.6)	103 (31.3)	0.15	23 (23.0)	0.05
Deep breathing	3 (33.3)	77 (23.4)	0.45	13 (13.0)	0.13
Hypoxia	1 (11.1)	30 (9.1)	0.59	12 (12.0)	0.99
**Hematological complications**	8 (88.9)	283 (86.0)	0.99	84 (84.0)	0.99
Severe anemia [a]	5 (55.6)	111 (33.8)	0.28	35 (35.4)	0.29
Thrombocytopenia [b]	5 (55.6)	242 (73.8)	0.26	67 (67.7)	0.48
Blackwater fever	1 (11.1)	72 (22.0)	0.69	21 (21.4)	0.68
**Hepatic complications**	7 (77.8)	220 (66.9)	0.72	65 (65.0)	0.72
Jaundice	3 (33.3)	71 (21.6)	0.42	21 (21.0)	0.41
Hypoalbuminemia [c]	6 (66.7)	192 (58.7)	0.74	58 (58.6)	0.74
**Kidney complications**	5 (55.6)	170 (51.7)	0.99	48 (48.5)	0.74
AKI [d]	5 (55.6)	150 (45.6)	0.74	44 (44.4)	0.73
Severe AKI [e]	2 (22.2)	63 (19.1)	0.69	20 (20.2)	0.99
**Metabolic complications**					
Acidosis	4 (44.4)	164 (49.8)	0.99	47 (47.0)	0.99
Hypoglycemia [f]	1 (11.1)	12 (3.7)	0.30	6 (6.1)	0.47
Hyponatremia [g]	2 (22.2)	27 (8.8)	0.20	8 (8.6)	0.21
Hyperkalemia [h]	2 (22.2)	16 (5.2)	0.09	9 (9.8)	0.25
**Comorbidities**					
HIV infection	1 (12.5)	8 (2.4)	0.20	3 (3.0)	0.27
Bacteremia	0	10 (3.1)	.	4 (4.1)	0.99
**Spleen**					
Spleen palpable	4 (44.4)	121 (36.8)	0.73	61 (61.0)	0.74

**Intestinal injury markers**					
TFF3, median (IQR), ng/mL	2.8 (2.4, 3.3)	2.3 (1.6, 3.4)	0.46	2.1 (1.6, 2.9)	0.31
I-FABP, median (IQR), ng/mL	2.1 (1.8, 7.7)	2.4 (1.3, 4.2)	0.56	2.0 (1.2, 3.3)	0.38
**Parasite factors**					
PfHRP2, median (IQR), ng/mL	1292 (598, 1820)	3528 (1364, 7365)	0.06	3344 (1643, 6638)	0.07
Parasite density, median (IQR) parasites/μL	15009 (4937, 41021)	64169 (8726, 249278)	0.12	27611 (2427, 150613)	0.53

One sample with *Pm* single infection and one sample with *Pf* and *Pm* mixed infections are not represented in this table. Values are presented as n (%) except where noted. Fisher's exact test used to compare proportion with or without condition; Wilcoxon rank-sum test used to compare continuous variables. Abbreviations: AKI, acute kidney injury; IQR, interquartile range.

For these secondary analyses, *P* values were not adjusted for multiple comparisons.

[a] Hemoglobin ≤ 5 g/dL.

[b] Platelet count < 150 × 10^3/μL.

[c] Albumin < 3.5mg/dL.

[d] AKI, 1.5-fold increase in serum creatinine from estimated baseline or 0.3 mg/dL increase in creatinine within 24 hours of admission.

[e] Severe AKI, ≥ 3.0-fold increase in serum creatinine over baseline.

[f] Glucose < 2.2 mmol/L on admission.

[g] Hyponatremia defined as a sodium < 130 mmol/L on admission.

[h] Potassium ≥ 6.0 mmol/L.

Table S3. Blood culture results in children with severe malaria, according to *Plasmodium* species infection

	***Pf* + *Po* + *Pm* (n=8)** [a]	***Pf* only (n=320)**	***Pf* + *Po* (n=98)**	***P*-value**
**Blood Culture negative**, n (%)	8 (100)	311 (97)	94 (96)	0.99 [b]
**Blood Culture positive**, n (%)	0	9 (3)	4 (4)	
Staphylococcus aureus	0	2	1	
Salmonella, non-typhi	0	1	0	
Other gram-negative b	0	3	1	
Other gram-positive b	0	0	0	
Enterococcus	0	1	1	
Proteus mirabilis	0	1	0	
Salmonella, typhi	0	1	0	
Escherichia coli	0	0	1	

Values are presented as n (%). Fisher's exact test used to compare proportion. [a] one child did not have sample for blood culture testing. [b] Pairwise comparisons revealed no two groups differed. Abbreviations: *Pf, P. falciparum*; *Pm*, *P. malariae; Po, P. ovale.*
